# Dimethyl Fumarate and Monomethyl Fumarate Promote Post-Ischemic Recovery in Mice

**DOI:** 10.1007/s12975-016-0496-0

**Published:** 2016-09-10

**Authors:** Yang Yao, Weimin Miao, Zhijia Liu, Wei Han, Kaibin Shi, Yi Shen, Handong Li, Qiang Liu, Ying Fu, DeRen Huang, Fu-Dong Shi

**Affiliations:** 1 Department of Neurology, Tianjin Neurological Institute, Tianjin Medical University General Hospital, Tianjin, 300052 China; 2 The State Key Laboratory of Experimental Hematology, Chinese Academy of Medical Sciences and Peking Union Medical College, 288 Nanjing Road, Tianjin, 300020 China; 3 Department of Radiology, Tianjin Neurological Institute, Tianjin Medical University General Hospital, Tianjin, 300052 China; 4 Department of Neurology, Barrow Neurological Institute, St. Joseph’s Hospital and Medical Center, Phoenix, AZ 85013 USA; 5 Neurology and Neuroscience Associates, Unity Health Network, Akron, OH USA

**Keywords:** Dimethyl fumarate, Monomethyl fumarate, Stroke, Nrf2, HO-1

## Abstract

Oxidative stress plays an important role in cerebral ischemia–reperfusion injury. Dimethyl fumarate (DMF) and its primary metabolite monomethyl fumarate (MMF) are antioxidant agents that can activate the nuclear factor erythroid-2-related factor 2 (Nrf2)/heme oxygenase-1 (HO-1) pathway and induce the expression of antioxidant proteins. Here, we evaluated the impact of DMF and MMF on ischemia-induced brain injury and whether the Nrf2 pathway mediates the effects provided by DMF and MMF in cerebral ischemia–reperfusion injury. Using a mouse model of transient focal brain ischemia, we show that DMF and MMF significantly reduce neurological deficits, infarct volume, brain edema, and cell death. Further, DMF and MMF suppress glial activation following brain ischemia. Importantly, the protection of DMF and MMF was mostly evident during the subacute stage and was abolished in Nrf2^−/−^ mice, indicating that the Nrf2 pathway is required for the beneficial effects of DMF and MMF. Together, our data indicate that DMF and MMF have therapeutic potential in cerebral ischemia–reperfusion injury and their protective role is likely mediated by the Nrf2 pathway.

**Electronic supplementary material:**

The online version of this article (doi:10.1007/s12975-016-0496-0) contains supplementary material, which is available to authorized users.

## Introduction

Stroke is the second leading cause of death globally and composed of hemorrhagic and ischemic stroke, with the latter being more common and resulting from disruption of blood supply to the brain [1, 2]. Thrombolysis with tissue plasminogen activator (tPA) and mechanical thrombectomy [3, 4] is effective at recanalization of an occluded artery, thus restoring cerebral circulation. However, reperfusion upon spontaneous or medically induced recanalization after cerebral ischemia can cause tissue injury which in turn contributes to worsening neurological status as well as increased morbidity and mortality in patients with acute ischemic stroke [5]. Reperfusion injury consists of a multi-step cascade with a wide range of mechanisms, including disturbance of protein synthesis, oxidative stress, platelet activation, inflammatory immune responses, disruption of the blood–brain barrier (BBB), glial activation, and neuronal apoptosis [6–8]. Some pharmacological agents have been studied to address ischemia–reperfusion injury by blocking reactive oxygen species (ROS) and neuronal excitotoxicity [9, 10]. However, these agents have thus far failed to demonstrate efficacy in clinical trials [11, 12]. The complexity of the ischemia–reperfusion biological cascade, inadequate dosing of antioxidants, inappropriate targeting by antioxidants, and most importantly the narrow time window (from minutes to approximately 3 h) for targeting oxidative stress in clinical settings are among the possible reasons that have led to the failure of these clinical trials [13–15]. It remains an urgent need to develop neuroprotective strategies targeting multiple key steps in the biochemical cascade of ischemia–reperfusion injury.

Dimethyl fumarate (DMF), derived from fumaric acid esters (FAE), represents a class of molecules exhibiting a multitude of biological effects including anti-oxidative stress and anti-apoptotic and immunomodulatory properties as well as providing protection from microvascular dysfunction in a variety tissues [16]. DMF exerts immunomodulatory effects on T cell subsets, glial cells, via the reduction of proinflammatory cytokines such as IL-2, TNF-α, and ICAM in inflammatory cascades. DMF stabilizes and activates the transcription factor nuclear factor (erythroid-derived 2)-like 2 (Nrf2), regulating many target genes such as HO-1, quinone oxidoreductase 1 (NADPH), and nuclear factor kappa-light-chain-enhancer of activated B cells (NF-kB) [17, 18]. DMF is approved for the treatment of relapsing multiple sclerosis in the US and European countries.

In a Parkinson’s disease model, DMF has been shown to ameliorate dopaminergic neurotoxicity [19]. In intracerebral hemorrhage animal models, DMF induced Nrf2 target genes, reduced cerebral edema and inflammation, improved hematoma resolution, and enhanced neurological recovery [20, 21]. Using a preconditioning, acute ischemic stroke model, Kunze et al. demonstrated that prophylactic treatment with DMF did not change the size of cerebral infarct, but was able to attenuate edema formation [22]. The unpredictable onset of stroke extremely limits the prophylactic use of DMF in clinical practice. The immediate impact of DMF on acute ischemic stroke and its post-conditioning role in treating acute ischemic stroke have not been assessed.

In the present study, we examined a potential therapeutic role for DMF in the acute and subacute stages following middle cerebral artery occlusion (MCAO) and reperfusion injury. Given the significant biological difference between DMF and its major metabolite, monomethyl fumarate (MMF) [23, 24], comparisons between DMF and MMF were carried out. Our data demonstrated that DMF and MMF exert their protective role by reducing infarct size during the subacute stage of stroke, but not immediately following MCAO. This protection is likely due to increased Nrf2 activity.

## Materials and Methods

### Animals

Nrf2 knockout mouse line was generously provided by Dr. Thomas W. Kensler of the University of Pittsburgh [25]. Mice were backcrossed to the C57BL/6 background for more than 10 generations. Heterozygous (Nrf2^+/−^) mice were used to produce homozygous (Nrf2^–/–^) and wild-type (WT) littermates. Animals (20 to 25 g, 8 to 10 weeks old) had access to food and water ad libitum and were housed under controlled conditions (23 ± 2 °C, 12-h light/dark periods). Adequate measures were taken to minimize the number of experiment animals used and to ensure minimal pain or discomfort in animals. All mice were randomly assigned to the different experimental groups. Animal exclusion criteria were as follows: mice died within the observation period and subarachnoid or intracerebral hemorrhage macroscopically or by magnetic resonance imaging were excluded. All animal experiments and procedures were approved by the Animal Experiments Ethical Committee of Tianjin Medical University General Hospital.

### Middle Cerebral Artery Occlusion Model

Focal cerebral ischemia was modeled by occluding the left middle cerebral artery (MCAO), based on the methods described by Longa et al. [26]. Briefly, the mice were anesthetized with chloral hydrate (30 mg/kg, intraperitoneal injection). A midline neck incision was then made to expose the left common carotid artery, the external carotid artery, and the internal carotid artery, which were all then isolated and ligated. A monofilament coated with silicone rubber (Xinong, 1418A, Beijing, China) was inserted into the internal carotid artery (9–10 mm) through the common carotid artery, to the beginning of the middle cerebral artery (MCA). A laser Doppler approach was used to monitor MCA occlusion and reperfusion as we previously described [27]. For this procedure, a small incision was made in the skin overlying the temporalis muscle and a 0.7-mm flexible laser Doppler probe (model P10) was positioned on the superior portion of the temporal bone (6 mm lateral and 2 mm posterior from the bregma). One hour after the induction of ischemia, the monofilament was removed to restore blood flow. Relative cerebral blood flow had to rise to at least 50 % of preischemic levels for the mice to be included in the study and subjected to further analyses. The body temperature of the mice was maintained at 37.0 ± 0.5 °C during surgery, and the mice were kept in a well-ventilated room at 25 ± 3 °C in individual cages, with the provision of food and water, until they regained full consciousness.

### DMF and MMF Administration

**Fig. 1:**
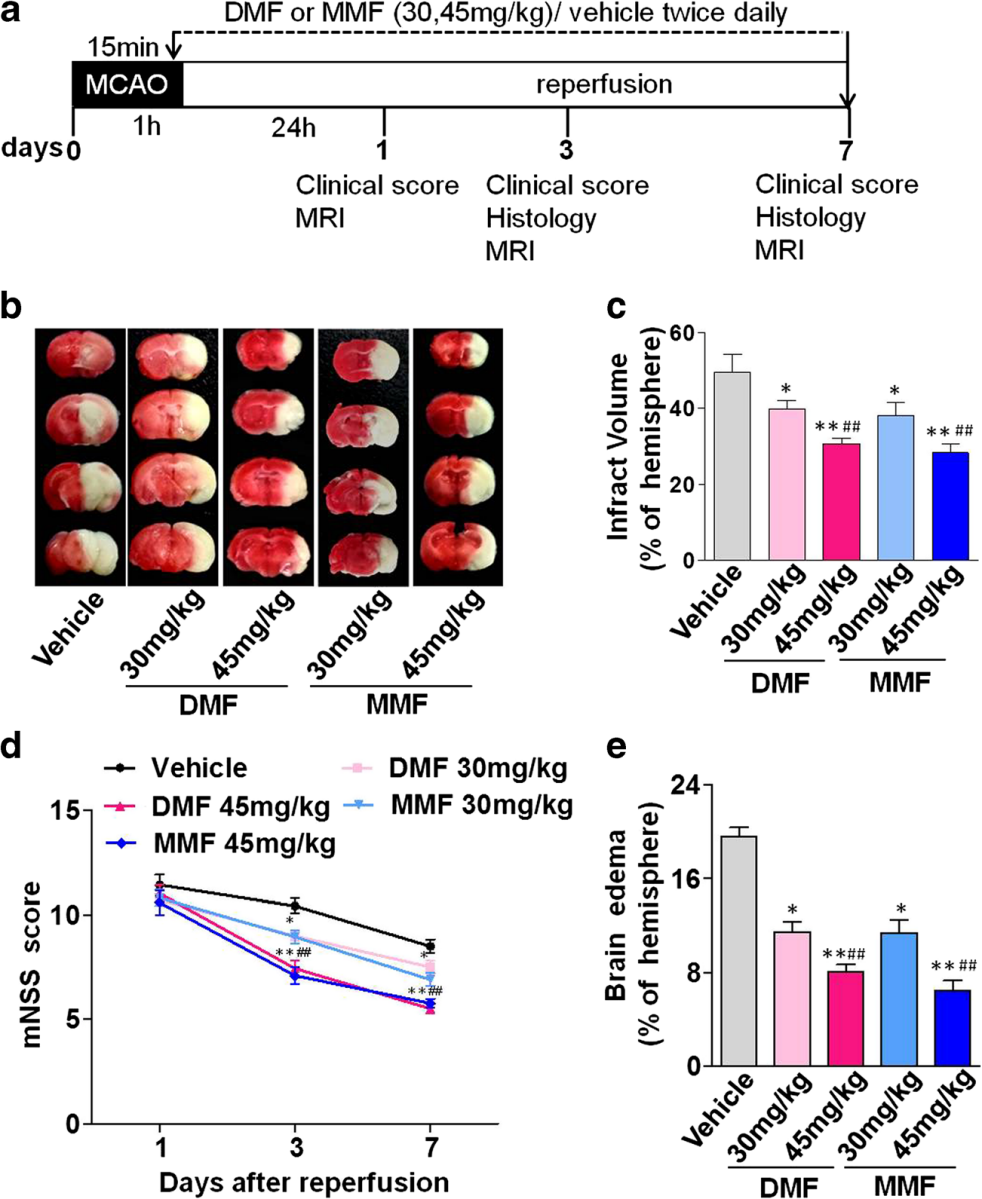
DMF and MMF improve neurological deficits and reduce brain infarct volume and cerebral edema in mice with MCAO. **a** Schematic experimental design of DMF and MMF treatment for acute ischemic cerebral stroke. **b** Administration of DMF or MMF reduces infarct volume determined by 2,3,5-tripenyltetrazolium chloride (TTC) staining. Brain sections from a representative mouse are shown from each group (vehicle control, DMF 30, 45 mg and MMF 30, 45 mg). Each *column* shows four TTC-stained coronal brain slices arranged in cranial to caudal order. **c** DMF and MMF treatments reduce the infarct volume due to MCAO during the subacute phase. Mice in the DMF- and MMF-treated groups had smaller volume of infarct on days 3 and 7 post-ischemia–reperfusion injury. Data shown are mean ± SEM at day 3; *p < 0.05, **p < 0.01 as compared to the vehicle-treated group; ##*p* < 0.01, as compared between the DMF 45 mg group and DMF 30 mg group, or MMF 45 mg and MMF 30 mg group, *n* = 10 per group. **d** Neurobehavioral improvement in mice with MCAO upon DMF or MMF treatment is time- and dose-dependent. Significant improvement in mNSS scores is evident in mice treated with 45 mg/kg DMF or MMF (*p* < 0.01) as compared to mice in the vehicle control group on day 3 and day 7, but not on day 1 post-ischemia–reperfusion injury. A lesser degree of improvement is observed in mice treated with DMF or MMF at a dose of 30 mg/kg body weight. Mean ± SEM; **p<0.01, as compared to those in the vehicle control group. **e** DMF and MMF attenuate cerebral edema associated with transient MCAO in a dose-dependent manner. Data depicted here are mean ± SEM; *p < 0.05 and **p < 0.01 as compared to the vehicle-treated group; ##*p* < 0.01 as compared between DMF 45 mg/kg and DMF 30 mg/kg group or MMF 45 mg/kg and MMF 30 mg/kg group; *n* = 10 per group

DMF (Sigma-Aldrich, Steinheim, Germany) was dissolved in 10 % dimethyl sulfoxide (DMSO). Given the evidence of a dose-dependent effect in antioxidant strategy in general [13] and with DMF [28–30], we performed dose-finding experiments with oral gavage administration of DMF in a mouse MCAO model (Supplemental Fig. 1). Subsequently, DMF was given at 30 or 45 mg/kg body weight, twice a day, for seven consecutive days with the first dose given 15 min before reperfusion (Fig. 1a). MMF (Sigma-Aldrich, Steinheim, Germany) was dissolved in 0.01 M phosphate-buffered saline (PBS) and administered intraperitoneally (i.p.) at a dosage of 30 or 45 mg/kg body weight, twice a day, for seven consecutive days with the first dose given 15 min before reperfusion (Fig. 1a).

To discern a potential post-ischemic role for both DMF and MMF, WT mice were divided into five groups: vehicle (MCAO + PBS, *n* = 50), DMF 30 mg/kg (MCAO + DMF 30 mg/kg, *n* = 50), DMF 45 mg (MCAO + DMF 45 mg/kg, *n* = 50), MMF 30 mg/kg (MCAO + MMF 30 mg/kg, *n* = 50), and MMF 45 mg/kg (MCAO + MMF 45 mg/kg, *n* = 50). Assessments included neurobehavioral testing and infarct volume assessment using MRI in vivo and 2,3,5-tripenyltetrazolium chloride (TTC) staining in vitro at days 1, 3, and 7 post-ischemia as well as brain edema and tissue pathology (Fig. 1a)

To test whether the Nrf2/HO-1 pathway mediates the effects provided by DMF and MMF in cerebral ischemia reperfusion injury, Nrf2^−/−^ mice with MCAO were randomly divided into three groups: control (Nrf2^−/−^ + PBS, *n* = 18), DMF 45 mg/kg (Nrf2^−/−^ + DMF 45 mg/kg, *n* = 18), and MMF 45 mg/kg (Nrf2^−/−^ + DMF 45 mg/kg, *n* = 18). Assessments included neurobehavioral testing as well as brain edema and tissue pathology assessment (Fig. 1a).

The arbitrary time points after MCAO in this study represent the following stages of stroke [27, 31]: <1 day, acute stage; 2–7 days, subacute stage.

### Neurobehavioral Monitoring

A battery of neurobehavioral tests was performed before MCAO and on days 1, 3, and 7 after MCAO induction by two investigators who were blinded to the experimental group assignment. These tests were summarized and expressed as the modified neurological severity score (mNSS), a composite of motor, sensory, reflex, and balance tests. Neurological function was graded on a scale of 0–18 as previously described [32], with the higher score, the more severe impairment from the ischemia–reperfusion injury.

### 2,3,5-Tripenyltetrazolium Chloride Assessment of Infarct Size

Brain tissues of mice were obtained at days 1, 3, and 7 post-ischemia and immediately sliced into coronal sections (2 mm thick) from the rostral to the caudal frontal tip using scalpels. The sections were stained with 1.5 % TTC (Sigma-Aldrich, USA), followed by immersion in normal saline at 37 °C for 20 min. Brain sections were then fixed in 4 % paraformaldehyde at 4 °C overnight before being photographed. With this staining method, viable tissues stain deep red based on intact mitochondrial function, while infarcts remain white. The infarcted regions in each section were evaluated using Image-Pro® Plus v 4.0 image analysis software (Media Cybernetics, Washington, DC, USA). The total infarct volume was calculated as the sum of the infarct volume of each section. The infarct volume percentage was calculated as follows: ([total contralateral hemispheric volume] − [total ipsilateral hemispheric stained volume])/(total contralateral hemispheric volume) × 100 % [33].

### Magnetic Resonance Imaging

Magnetic resonance imaging (MRI) data were acquired using a 3.0-Tesla clinical MR system (Discovery MR750, General Electric, Milwaukee, WI, USA), using an eight-channel phased-array head coil. Coronal T2-weighted turbo spin echo (10 continuous slices, repetition time (TR) = 2980 ms, echo time (TE) = 78 ms, section thickness = 1 mm, in-plane resolution 1 mm^2^) was used for lesion detection. The lesion borders on each slice were traced manually by using Image-Pro Plus (Media Cybernetics, Rockville, MD, USA) to measure the lesion area, then summed and multiplied by the slice thickness to determine the lesion volume.

### Brain Edema Measurement

At day 3 after MCAO, mice were sacrificed and brains were harvested. Each brain was carefully divided into three parts as left hemisphere, right hemisphere, and cerebellum. Tissues were quickly weighed on an electronic analytical balance to obtain the wet weight. The brain tissues were then dried for 72 h at 100 °C to obtain the dry weight. Brain water content calculation was achieved using the following formula: (wet weight − dry weight) / wet weight × 100 %.

### Immunostaining

The extent of cell death was assessed using a terminal deoxynucleotidyl transferase biotin-dUTP nick end labeling (TUNEL) kit (Roche, USA). Once stained, the specimens were analyzed under a florescence microscope (Nikon C-HGFI, Japan). The total number of nuclei and TUNEL-positive cells were counted in four random fields of a ×20 view of the edge of the infarct, and the ratio of apoptotic cells to nuclei was calculated as apoptotic cells in percent. Paraffin sections from mouse brains sacrificed 24 h after MCAO, cut to a thickness of 5 μm, were first deparaffinized in xylene and subsequently rehydrated with various grades of ethanol. After rehydrating the sections, nonspecific binding was blocked by incubating the sections in 10 % bovine serum albumin for 20 min. The sections were then incubated overnight at room temperature with anti-glial fibrillary acidic protein (GFAP) or anti-ionized calcium-binding adapter molecule (Iba-1) antibodies to identify astrocytes and microglia, respectively. Finally, mounting media containing DAPI was applied and a coverslip was placed over the sections. The stained sections were examined and analyzed with a fluorescence microscope (Olympus, Tokyo, Japan).

### Determination of Indicators of Oxidative Stress

Brain tissues were collected at day 3 after MCAO. Superoxide dismutase (SOD) activity as well as the levels of malondialdehyde (MDA) and glutathione (GSH) were measured as indicators of oxidative stress [34]. The brains were washed, weighed, and then homogenized in ice-cold saline (nine volumes) for 20 min to prepare a 10 % (*w*/*v*) homogenate. The homogenate was then centrifuged at 4000 rpm/min for 10 min at 4 °C. SOD activity and levels of MDA and GSH were measured as described previously [34, 35]. Data were calculated in reference to the protein concentration in each sample.

### Western Blot Analysis

On day 3 post-MCAO, ipsilateral hemispheres were homogenized in RIPA lysis buffer (Sigma, USA) and 1 mmol/L phenylmethanesulfonyl fluoride (PMSF) (Sigma, USA). After centrifugation, the supernatants were collected as total proteins. Proteins were loaded and transferred to a PVDF membrane (Millipore, USA). After being blocked, membranes were incubated overnight at 4 °C with anti-Nrf2 (1:1000, Millipore), anti-HO-1 (1:1000, Millipore), or rabbit polyclonal antibodies. Membranes were then incubated for 1 h at room temperature with horseradish peroxidase (HRP)-labeled goat anti-rabbit secondary antibody (1:4000, Vector, Burlingame, USA). The membranes were placed into a gel imaging system (Bio-Rad, ChemiDoc XRS, USA) and then exposed. The intensity of blots was quantified using the Quantity One Analysis software (Bio-Rad, USA). β-Actin was used as an internal control.

### RNA Isolation and Real-Time PCR

Total RNA was extracted from the ischemic hemisphere using TRIzol Reagent (Invitrogen, Carlsbad, CA, USA) according to the manufacturer’s instructions at day 3 after MCAO. The concentration of RNA was quantified by ultraviolet spectrophotometry at 260/280 nm. cDNA was transcribed using TransScript First-Strand cDNA Synthesis SuperMix Kit (TransGen; catalog no. AT301) in accordance with the manufacturer’s instructions. PCR was performed on the Opticon 2 Real-Time PCR Detection System (Bio-Rad) using the following primers: Nrf2 forward GGTTGCCCACATTCCCAAAC, Nrf2 reverse TCCTGCCAAACTTGCTCCAT; HO-1 forward CGACAGCATGTCCCAGGATT, HO-1 reverse CTGGGTTCTGCTTGTTTCGC; and β-actin forward AAATCGTGCGTGACATCAAAGA, β-actin reverse GGCCATCTCCTGCTCGAA; SYBR Green PCR Master Mix (Roche) was also used. Samples were run in duplicate and normalized to β-actin using the 2^–ΔΔCt^ method. The expression levels of the messenger RNAs (mRNAs) were then reported as fold changes vs control.

### Statistical Analysis

All data were analyzed by SPSS 18.0 software and expressed as mean ± SEM. Sample size per group was determined using a priori sample size calculation (G*Power version 3.1). To achieve *α* = 0.05 at *β* = 0.2 (power 80 %) with a mean 20 % standard deviation, results from sample size calculation show that *n* = 6–10 mice per group was appropriate. Statistical differences were measured by unpaired two-tailed Student *t* test for comparison of two groups or ANOVA followed by Bonferroni post hoc test for multiple group comparisons. Values of *p* < 0.05 were considered significant.

## Results

### Improvement in Neurological Deficits at Subacute Stage in MCAO Mice Treated with DMF or MMF

On day 1 post-MCAO ischemia–reperfusion injury, there was no statistical difference in mNSS between groups of MCAO mice treated with vehicle control, DMF (30, 45 mg/kg), or MMF (30, 45 mg/kg) groups. Surprisingly, on day 3 post-MCAO ischemia–reperfusion injury, mice in both of the DMF (30 and 45 mg/kg)-treated groups exhibited significantly reduced mNSS. A similar impact was evident in mice receiving MMF (30 and 45 mg/kg) treatment. The improvement of neurobehavioral function with DMF or MMF treatment was dose-dependent with a better outcome in mice treated with the relatively higher dose of DMF (45 mg/kg) or MMF (45 mg/kg) as compared with those treated with the lower dose of DMF (30 mg/kg) or MMF (30 mg/kg) body weight, respectively (Fig. 1d). Further, the reduction of mNSS was sustained on day 7 post-MCAO ischemia–reperfusion injury.

### DMF and MMF Reduce Infarct Volume

The infarct volume of brain tissue was measured on days 1, 3, and 7 post-MCAO ischemia–reperfusion injury with TTC staining (Fig. 1b). On day 1 post-MCAO, there was no statistical difference in the volume of the infarct in mice treated with vehicle and DMF (30, 45 mg per kg body weight) (Supplemental Fig. 2). However, a significant reduction of infarct volume was observed in MCAO mice treated with DMF on day 3 post-MCAO. The reduction of infarct volume with DMF treatment was sustained in mice on day 7 post-MCAO (Fig. 1b, c). A dose effect was observed with the higher dose of DMF at 45 mg/kg body weight having a greater effect on the infarct volume reduction. To further confirm the protective role of DMF, we examined its primary metabolite MMF. A similar impact on acute ischemic infarct volume was observed with MMF in a dose-dependent manner (Fig. 1b, c). Administration of DMF (30, 45 mg/kg) or MMF (30, 45 mg/kg) significantly decreased the percentage of the infarct volume from 49.36 ± 5.33 % in the vehicle group to 39.69 ± 6.65 % (*p* < 0.05), 30.64 ± 2.85 % (*p* < 0.01), 38.05 ± 3.60 % (*p* < 0.01), or 28.21 ± 2.58 % (*p* < 0.01) on day 7 post-ischemia–reperfusion injury, respectively.

### Brain Edema

The brain water content (BWC) in mice of the vehicle group was 19.6 ± 2.5 %, while the DMF-treated (30, 45 mg/kg) and MMF-treated (30, 45 mg/kg) groups had decreased BWC of 11.47 ± 2.3 % (*p* < 0.05, as compared with vehicle group), 8.1 ± 1.9 % (*p* < 0.01), 11.42 ± 3.52 % (*p* < 0.05), or 6.42 ± 2.51 % (*p* < 0.01), respectively (Fig. 1e).

### Longitudinal Examination of Infarct Volume with MRI

**Fig. 2:**
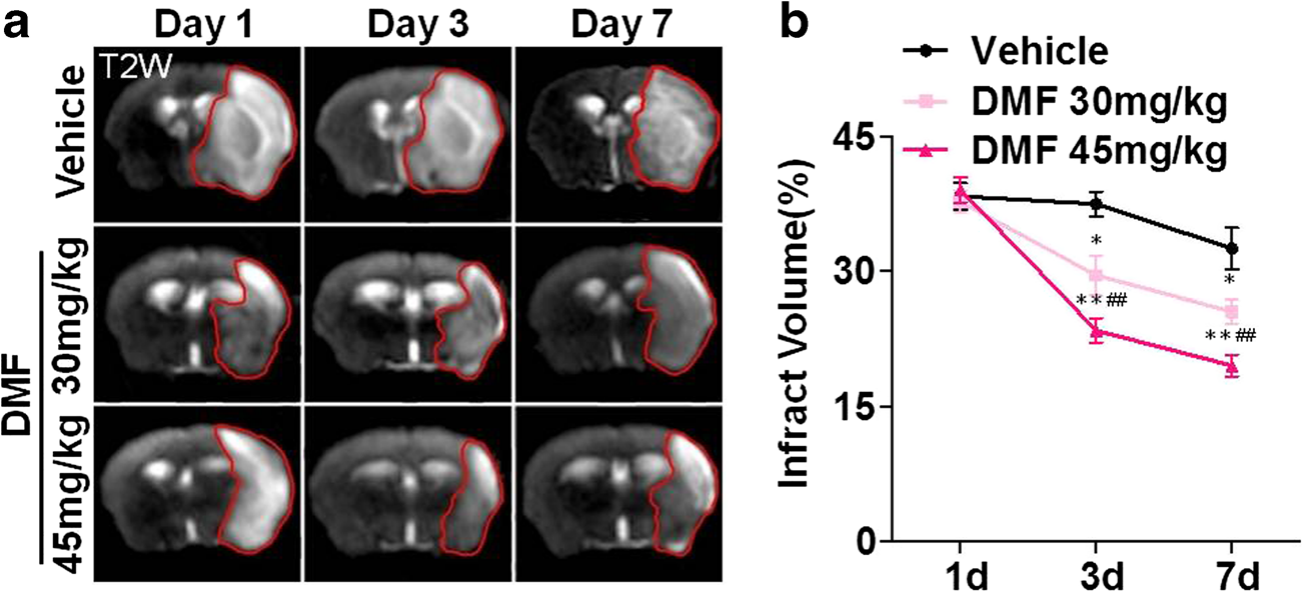
Effects of DMF on infarct volume were sustained. Coronal MRI sections show T2 lesions in vivo on days 1, 3, and 7 after MCAO. The volume reduction in ischemic lesions with DMF was sustained. Data shown are mean ± SEM; *p < 0.05, **p < 0.01 as compared to the vehicle group; #*p* < 0.05, ##*p* < 0.01, as compared between DMF 45 mg/kg and DMF 30 mg/kg group, *n* = 10 per group

To further monitor the evolution of ischemic brain lesions following MCAO ischemia–reperfusion injury, MRI was employed to image the lesions in vivo on days 1, 3, and 7 post-MCAO ischemia–reperfusion injury. T2-weighted images were used to calculate the volume of ischemic lesions. On day 1 post-MCAO ischemia–reperfusion injury, there was no difference in the volume of ischemic lesion in the vehicle-treated control group as compared to those in the DMF (30 mg or 45 mg/kg)-treated groups. However, on day 3 post-ischemia, the volume of ischemic lesion was significantly reduced in mice treated with DMF at a dose of 30 or 45 mg per kg body weight. The DMF-induced ischemic volume reduction on T2-weighted images was sustained on following MRI examinations at day 7 post-MCAO ischemia–reperfusion injury (Fig. 2).

### DMF and MMF Inhibit Neural Apoptosis

**Fig. 3:**
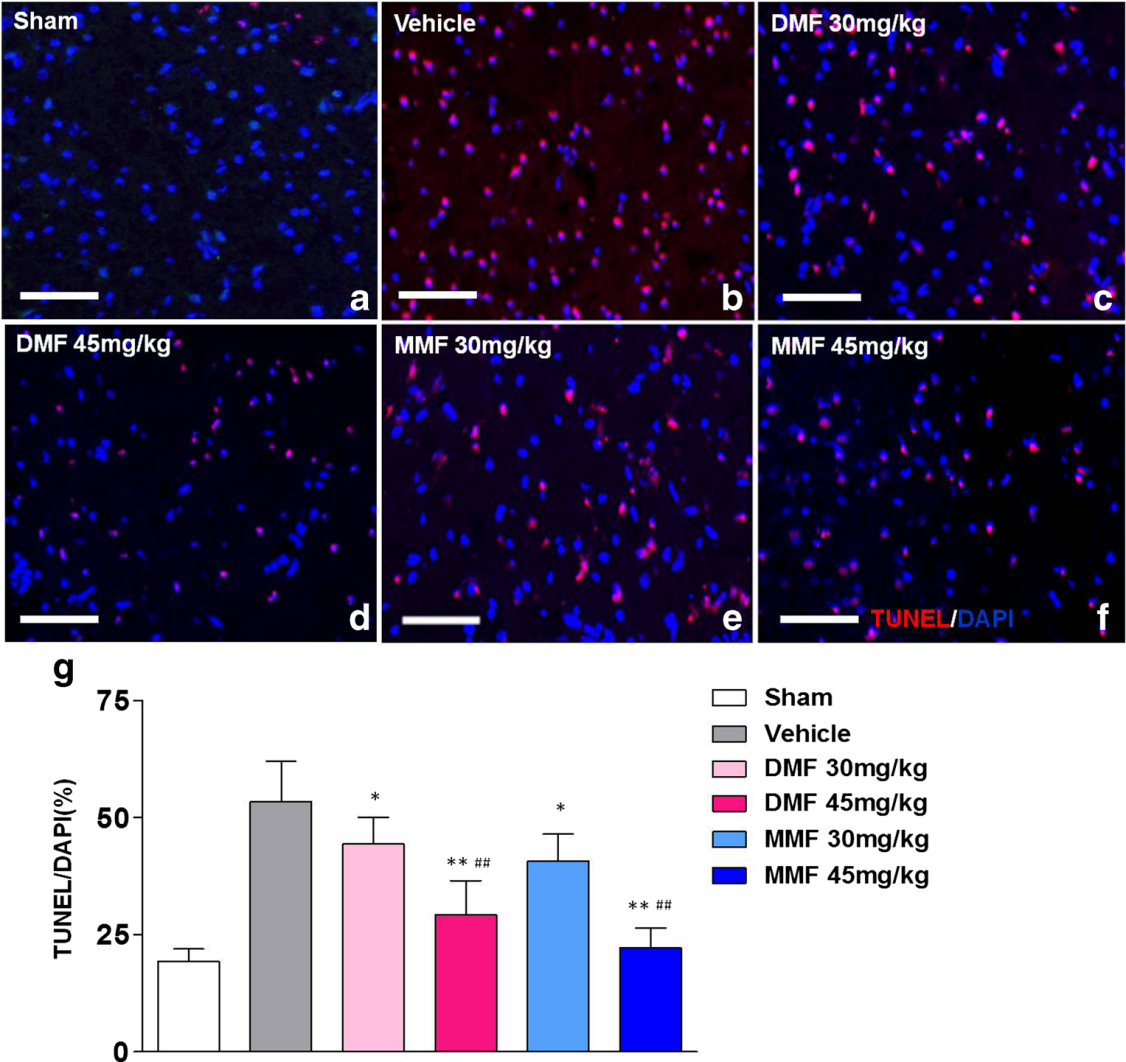
DMF and MMF reduce cell death caused by cerebral ischemic–reperfusion injury. TUNEL staining shows a large number of apoptotic cells in brain sections at 72 h post-ischemia–reperfusion injury. **a** In the vehicle-treated group, numerous TUNEL-positive cells are present in the ischemic cortex. **b**–**e** DMF or MMF treatment reduces the number of apoptotic cells. **f** Quantitative analysis of TUNEL-positive cells shows DMF- and MMF-induced protection from cell death and its dose-dependent pattern. Data are presented as mean ± SEM; *p < 0.05, **p < 0.01, compared to the vehicle group; ##*p* < 0.01, as compared between DMF 45 mg and DMF 30 mg group, or between MMF 45 mg and MMF 30 mg group; *n* = 8 per group

A TUNEL assay was used to assess neuronal apoptosis. TUNEL staining was performed in brain tissue sections obtained at day 3 after MCAO. In the sham group, no discernable neural apoptosis was seen. In the vehicle group, TUNEL-positive cells were densely distributed in the ischemic cortex (53.4 ± 2.61 %). The number of apoptotic cells was decreased in mice treated with DMF 30 mg (44.1 ± 2.64 %, *p* < 0.05 as compared to that in the vehicle-treated group), DMF 45 mg (29.2 ± 1.3 %, *p* < 0.01), MMF 30 mg (40.7 ± 0.77 %, *p* < 0.05), or MMF 45 mg/kg body weight (22.2 ± 2.12 %, *p* < 0.01). These results suggest a protective role of DMF or MMF against apoptosis of cells in the central nervous system (CNS) arising from ischemia–reperfusion injury (Fig. 3).

### DMF and MMF Treatment Leads to Suppressed Glial Activation

**Fig. 4:**
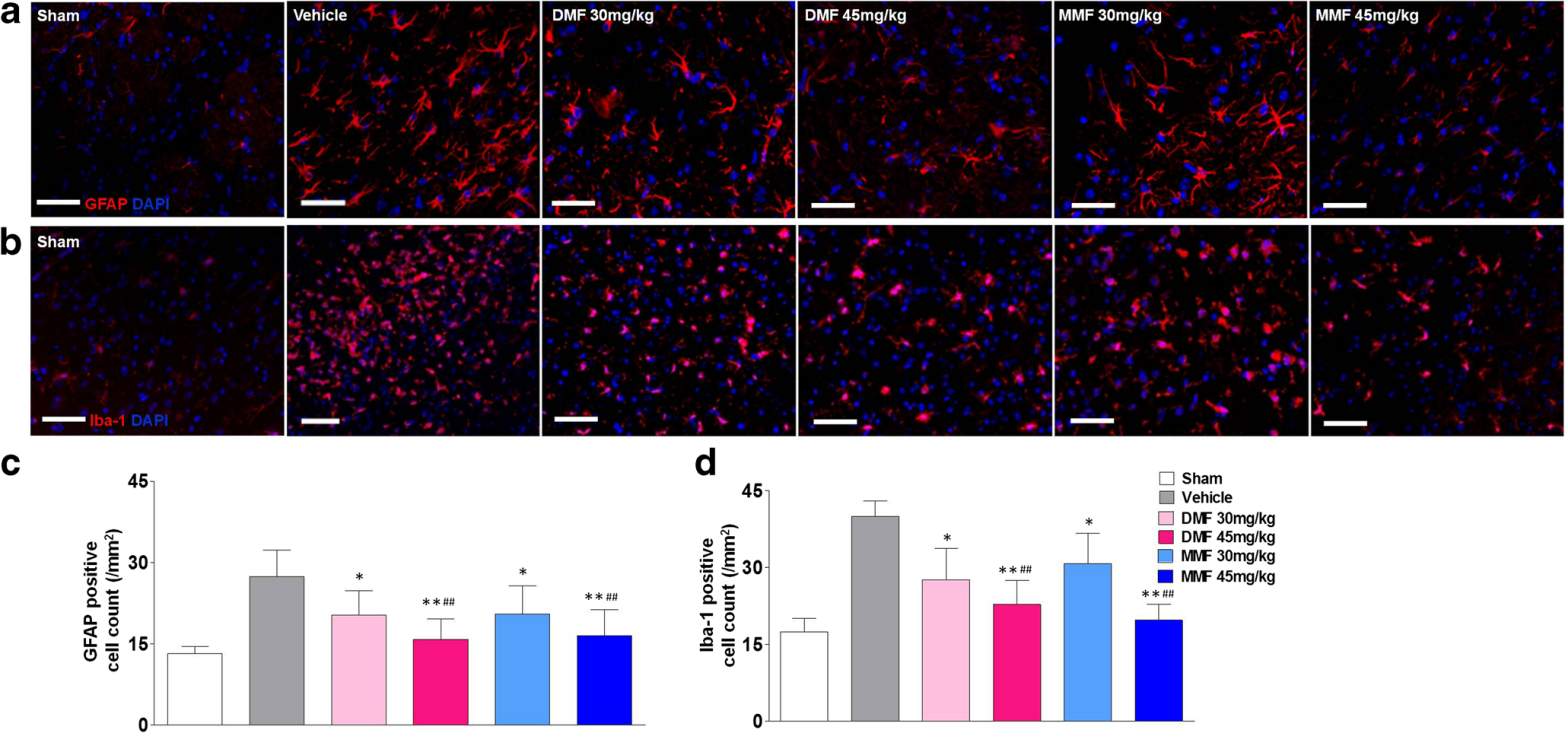
DMF or MMF suppresses glial activation associated with transient MCAO. **a** Brain slices from mice with MCAO stained with anti-glial fibrillary acidic protein (GFAP) antibody. **b** Brain slices stained with anti-ionized calcium-binding adapter molecule (Iba-1) antibody. Brain tissue sections obtained from mice 72 h after transient MCAO show increased astrocytosis and microgliosis (vehicle control in **a**, **b**, respectively). Treatment with DMF 30, 45 mg/kg or MMF 30, 45 mg/kg body weight reduces the expression of GFAP and Iba-1. **c** Quantitative analysis of GFAP-bearing cells and **d** that of Iba-1-bearing cells in cerebral tissues from mice with transient MCAO treated with DMF or MMF. The percentages of GFAP- and Iba-1-expressing cells are decreased by DMF or MMF treatment in a dose-dependent manner. Data are presented as mean ± SEM; *p < 0.05, **p < 0.01 as compared to the vehicle group; ##*p* < 0.01 as compared between DMF 45 mg vs DMF 30 mg or MMF 45 mg vs MMF 30 mg groups; *n* = 8 per group

Neural cell death in cerebral ischemia–reperfusion injury is associated with astrocytosis and microgliosis [36]. To test the hypothesis that cytoprotective properties of DMF and MMF might diminish the glial activation elicited by transient ischemia and reperfusion, immunohistochemistry studies were performed. As shown in Fig. 4, the numbers of GFAP-positive and Iba-1-positive cells were decreased in the ischemic region of the MCAO mice as compared to the sham group. Compared with the vehicle group (27.4 ± 2.90 %), the number of GFAP-positive cells was decreased in the DMF 30 mg (20.3 ± 2.47 %, *p* < 0.05), DMF 45 mg (16 ± 3.08 %, *p* < 0.01), MMF 30 mg (20.5 ± 2.23 %, *p* < 0.05), and MMF 45 mg/kg body weight (17 ± 2.71 %, *p* < 0.01) treated groups. The number of Iba-1-bearing cells was also decreased in the DMF 30 mg (27.6 ± 2.11 %, *p* < 0.05), DMF 45 mg (22.8 ± 2.7 %, *p* < 0.01), MMF 30 mg (29.8 ± 1.57 %, *p* < 0.05), and MMF 45 mg/kg body weight (19.7 ± 3.12 %, *p* < 0.01) treated groups as compared with the vehicle control group (40.0 ± 2.98 %) (Fig. 4).

### DMF and MMF Attenuate Ischemia–Reperfusion Injury Evoked Intracellular Oxidative Stress

SOD is often regarded as the first line of defense against an upswing of ROS and is responsible for the conversion of superoxide to H_2_O_2_ in the cytoplasm and mitochondria [37]. GSH plays a major role in the detoxification of peroxides [38]. Brain tissues with MCAO ischemia–reperfusion injury have decreased SOD activity and GSH levels. MDA, one of the products of membrane lipid peroxidation, reflects the degree of ROS-inflicted damage via membrane lipid peroxidation. Compared with the vehicle-treated group, there were significantly increased levels of SOD activity and GSH and decreased levels of MDA in the groups treated with DMF and MMF (30 and 45 mg/kg) (Table 1). These data suggest that DMF and MMF reduced MCAO-induced oxidative stress.

**Table 1:** Effects of DMF and MMF on transient MCAO-induced oxidative stress

Group	SOD (U/mg protein)	MDA (nmol/mg protein)	GSH (μmol/g protein)
Vehicle	92.04 ± 4.20	3.98 ± 0.65	8.54 ± 2.96
DMF 30 mg/kg	114.46 ± 6.57*	2.96 ± 0.58*	9.99 ± 2.91*
DMF 45 mg/kg	131.98 ± 6.13**^#^	2.18 ± 0.39**^#^	11.96 ± 2.16**^#^
MMF 30 mg/kg	109.98 ± 5.96*	2.99 ± 0.57*	10.07 ± 1.87*
MMF 45 mg/kg	125.46 ± 5.91**^#^	2.21 ± 0.51**^#^	11.64 ± 1.22**^#^

Data are presented as mean ± SEM. *n* = 8 per group

*DMF* dimethyl fumarate, *MMF* monomethyl fumarate, *MCAO* middle cerebral artery occlusion, *SOD* superoxide dismutase, *MDA* malondialdehyde, *GSH* glutathione

*p < 0.05; **p < 0.01, as compared to the vehicle group; #*p* < 0.01, comparisons between DMF 45 mg and DMF 30 mg group or MMF 45 mg and MMF 30 mg group

### DMF and MMF Promote Expression of Nrf2 and HO-1

**Fig. 5:**
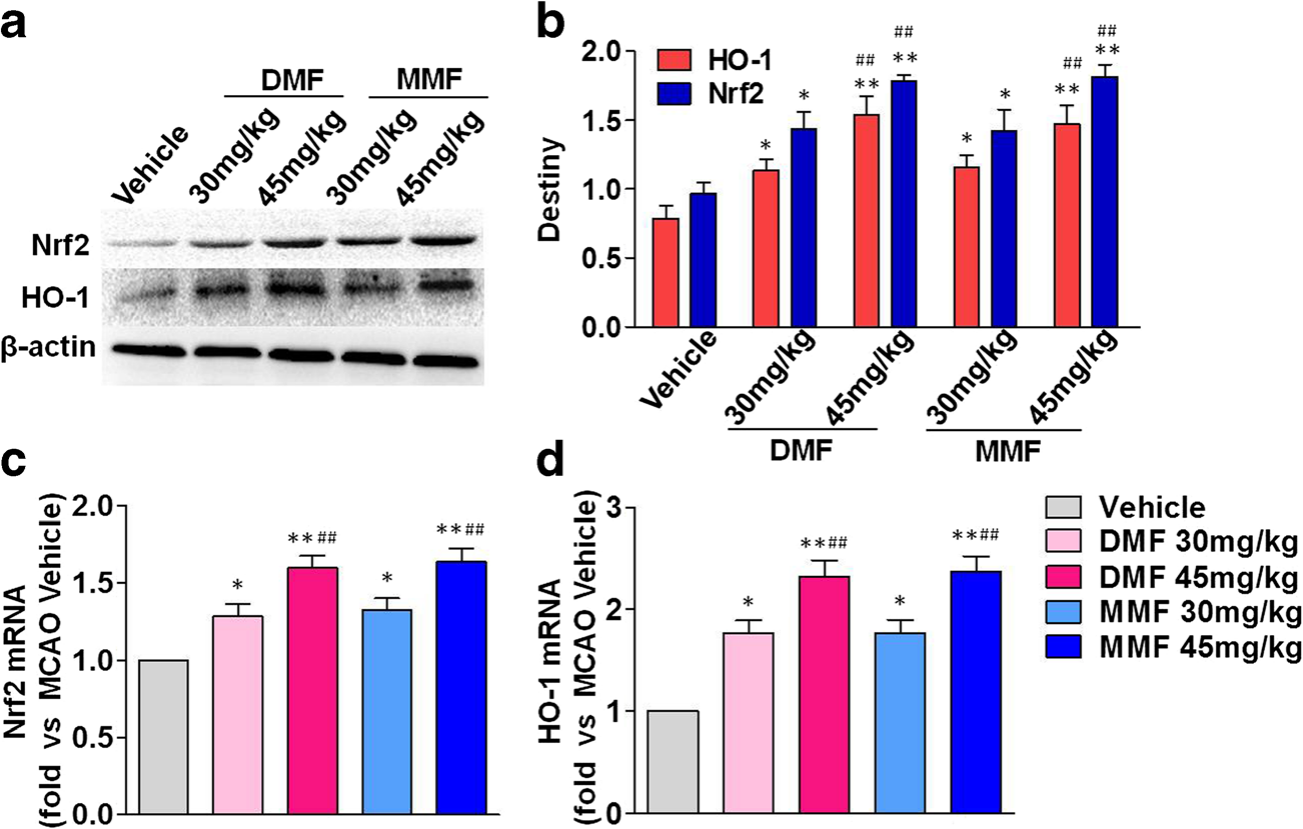
DMF and MMF promote the expression of Nrf2 and HO-1. **a**, **b** Western blot analysis of cortical tissues at 72 h after transient MCAO shows high levels of Nrf2 and HO-1 protein expression. DMF or MMF treatment increases levels of Nrf2 and HO-1 protein in mice with MCAO. **c**, **d** RT-PCR demonstrates that the levels of Nrf2 and HO-1 expression are upregulated by DMF and MMF treatment at 72 h. Data are presented as mean ± SEM; *p < 0.05, **p < 0.01 as compared to the vehicle control group; ##*p* < 0.01 as compared between DMF 45 and 30 mg/kg group or MMF 45 and 30 mg/kg group; *n* = 8 per group

To identify whether Nrf2 signaling is involved in the neuroprotective effect of DMF and MMF in MCAO ischemia–reperfusion injury, we analyzed ischemic brain tissues by western blot and RT-PCR. Western blot analysis of cortical tissues at day 3 post-ischemia reveals that DMF and MMF had no significant effects on Nrf2 levels in sham animals, but augmented the expression of Nrf2 and HO-1 protein upon DMF treatment in mice with MCAO. The induction of Nrf2 and HO-1 expression by DMF was further confirmed by RT-PCR analysis at the mRNA level. Moreover, increased levels of Nrf2 and HO-1 were evident in mice with MCAO treated with MMF (30 or 45 mg/kg) (Fig. 5).

### Absence of DMF or MMF Protection Against Ischemia–Reperfusion Brain Injury in Nrf2 Gene-Deficient Mice

**Fig. 6:**
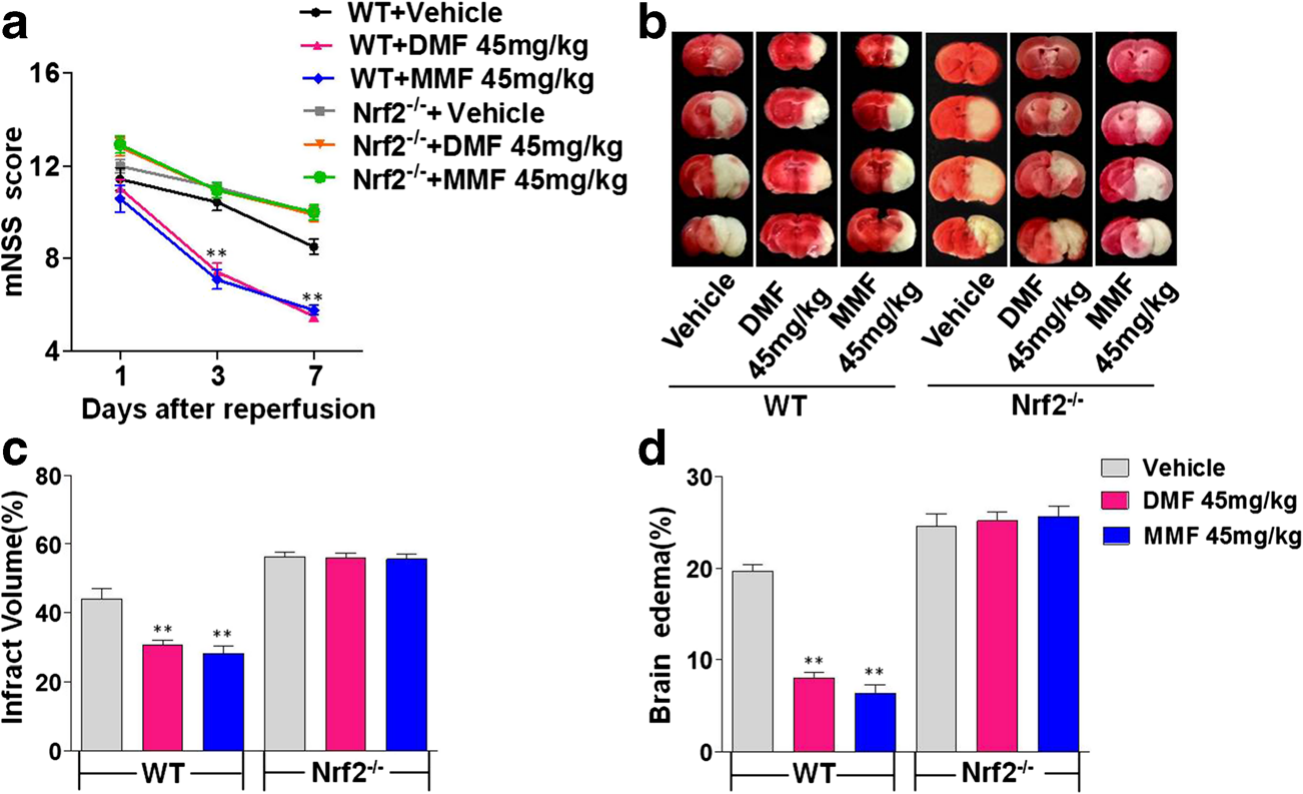
The neuroprotective effects of DMF and MMF are abolished in Nrf2^−/−^ mice. **a** The mNSS of DMF- or MMF-treated Nrf2^−/−^ mice with transient MCAO are comparable to those of vehicle-treated Nrf2^−/−^ mice on days 1, 3, and 7 post-ischemia-reperfusion injury. **b** TTC staining of cerebral tissue sections shows comparable sizes of infarction in Nrf2^−/−^ mice treated with DMF or MMF 45 mg/kg body weight. Note the reduction of infarct size in wild-type mice treated with DMF or MMF 45 mg/kg. **c** Quantitative analysis of infarct volume shows that DMF or MMF decreases infarct volume in wild-type but not in Nrf2-deficient mice on day 7 post-ischemia-reperfusion injury. Data are presented as mean ± SEM; **p < 0.01, compared to the vehicle WT group; *n* = 6 per group. **d** DMF or MMF treatment does not alter cerebral edema formation in Nrf2-deficient mice with MCAO ischemia-reperfusion injury. Note the significant reduction of cerebral edema in wild-type mice with transient MCAO upon DMF or MMF treatment. Data are presented as mean ± SEM; **p < 0.01, compared to the vehicle WT group; *n* = 6 per group

To further determine the mechanism by which DMF and MMF exert neuroprotective effects, Nrf2^−/−^ mice were used in the current study. Nrf2^−/−^ mice were treated with DMF (45 mg/kg) or MMF (45 mg/kg) after MCAO. There was no difference in infarct volume, mNSS, or brain edema between the vehicle and DMF 45 mg/kg-treated Nrf2^−/−^ groups. These findings were further confirmed with MMF 45 mg/kg treatment in Nrf2^−/−^ mice with MCAO (Fig. 6a–d). Furthermore, neither levels of HO-1 mRNA nor its protein expression was altered upon DMF or MMF treatment in Nrf2^–/–^ mice with MCAO ischemia–reperfusion injury (Supplemental Fig. 3).These findings suggest that the Nrf2 pathway is essential for DMF and MMF to exert their protective role in brain ischemia–reperfusion injury.

## Discussion

In the current study, we demonstrated that DMF and MMF exhibited neuroprotective properties against cerebral ischemia–reperfusion injury with faster and better recovery from initial ischemia–reperfusion insults. DMF or MMF treatment after transient focal cerebral ischemia resulted in improvement of neurological function coinciding with radiographic, histopathological, and biochemical changes. Interestingly, the neuroprotective properties of DMF and MMF were dose-dependent, which is consistent with human data generated in clinical trials in multiple sclerosis [28–30, 39].

The beneficial impact of DMF treatment initiated after the onset of cerebral ischemia and the comparable therapeutic effects from MMF treatment are plausible. The wide age range and random occurrence of acute ischemic stroke render prophylactic, chronic daily use of DMF unappealing [40, 41]. The gradual titration schedule and delayed-release form of DMF further complicates the application of DMF in acute stroke care. Parenteral administration of water-soluble fumarate compounds such as MMF might serve better for the treatment of acute ischemic stroke.

DMF has been studied and showed beneficial effects in a number of disease models [42–45]. The majority of DMF is rapidly metabolized to MMF in the intestine. It is difficult to trace in the systemic circulation though some DMF forms long-lived GSH conjugates [46, 47]. The concentration of the main metabolite MMF in the blood stream peaks at approximately 2–2.5 h. So far, the majority of mechanistic studies for fumarate compounds have been performed using DMF. Gillard et al. systematically analyzed DMF, MMF, and a mixture of monoethyl fumarate salts and showed a striking difference between DMF and MMF in various cellular responses in vitro. DMF but not MMF showed inhibition of NF-kB activity in an Nrf2-independent manner. In addition to the NF-kB pathway that modulates inflammatory immune response [48], other mechanisms of action related to DMF include the Nrf2-dependent antioxidant response pathway and functioning through a G-protein-coupled protein receptor, the hydroxycarboxylic acid receptor 2 (HCA2). In EAE, DMF reduces CNS recruitment of neutrophils via HCA2 [42, 49–53]. It is well known that infiltrating neutrophils peak within 24 h after ischemia–reperfusion [54]. Lack of protection against MCAO ischemia–reperfusion injury in DMF- or MMF-treated mice on day 1 post-ischemia suggests a minor role mediated by HCA2-dependent neutrophil infiltration.

Data from the current in vivo studies using the MCAO ischemia–reperfusion injury model demonstrated similar neuroprotective responses between DMF and MMF. The fact that both DMF- and MMF-induced neuroprotective effects were absent in Nrf2-deficient mice points toward a critical role of the Nrf2 pathway in cerebral ischemia–reperfusion injury. Nrf2 is a master regulator of the anti-oxidative defense responses [55–59], balancing between free radical and antioxidant defenses [60, 61]. Nrf2-deficient mice are more susceptible to oxidative stress [57]. Activation of the Nrf2 pathway is a promising therapeutic approach in neurodegenerative diseases [62, 63]. A recent study showed that S-allyl cysteine activates the Nrf2-dependent antioxidant response and protects neurons against ischemic injury [58]. Increased levels of SOD, HO-1, and GSH and decreased levels of MDA in DMF- or MMF-treated mice with MCAO further support the notion that DMF and MMF protect against cerebral ischemia–reperfusion injury and promote recovery via an Nrf2-dependent antioxidant pathway. The multiple actions of DMF or MMF allow all types of cells in the neurovascular unit to adapt to detrimental conditions after ischemia. The fast pharmacodynamics of MMF and the ester’s structure that enables rapid crossing of the blood–brain barrier to implement direct effects on CNS resident cells make MMF an attractive agent in treating acute ischemic stroke.

SOD and GSH are believed to be the critical scavenger enzymes hindering tissue injury caused by peroxidase reactions [34]. MDA, a toxic final product of lipid peroxidation, is inversely linked to the ability of SOD to reduce the rate and extent of lipid peroxidation free-radical productions [37]. NXY-059, a potent free radical-trapping agent, reduced size of cerebral infarct, improved recovery in early stages of development, and had significantly better outcome in the first phase 3 trial [64]. However, results from the 2nd phase III trial were neutral in the primary and all secondary outcome measurements [65]. It is believed that the discrepancy between experimental and clinical studies was attributable to the lack of long-term benefits in early experimental studies, protocol differences, in particular the timing and dosing of NXY-059, methodological quality, and poor permeability across the BBB [11, 12, 66]. Edaravone (MCI-186) is another free-radical scavenger [67] approved in Japan and recommended by the Japanese Guidelines for the Management of Stroke for acute ischemic stroke within 24 h from the onset of symptoms. The Japanese treatment protocol includes twice-a-day intravenous infusion of MCI-186 for a maximum of 14 days. Such a treatment protocol is not very practical due to requirement of prolonged admission in hospitals. On the other hand, restoring mitochondrial function and adequate supply of adenosine triphosphate (ATP) is essential for long-term neural tissue survival and normal brain function [14]. The reduced volume of TTC-stained brain tissue in DMF- or MMF-treated mice with MCAO and the gradual reduction of the area of hyperintense signal on T2-weighted images in the present studies are evident and clearly indicating the recovery of ATP supply after initial ischemia insult. The neuroprotective action is delayed, yet sustained. Therefore, promoting an endogenous, antioxidant Nrf2 pathway using DMF and MMF at the early stage of acute ischemic stroke is a novel strategy that deserves further exploration.

Evidence suggests that fumarates can stimulate the antioxidant response pathway via the activation of glial Nrf2 to suppress the production of inflammatory mediators [21, 22, 68–70]. In line with these previous reports, we found that DMF/MMF increased the expression of Nrf2 in MCAO mice and that the benefit of DMF/MMF requires Nrf2. Of note, the precise molecular mechanisms leading to fumarate-mediated activation of Nrf2 are not entirely understood. Reportedly, evidence suggests that fumarates can activate the Nrf2 pathway by direct modification or succination of cysteine residues in Keap1, which sequesters and degrades Nrf2 in the cytosol, leading to constitutive degradation [68, 71, 72]. Nevertheless, the necessity and sufficiency of modification of Keap1 for the fumarate-mediated activation of Nrf2 in the setting of ischemic stroke remain unclear and warrant further investigations.

## Electronic supplementary material

Below is the link to the electronic supplementary material.

ESM 1 (DOCX 123 kb)

## Abbreviations

DMF: Dimethyl fumarate
MMF: Monomethyl fumarate
Nrf2: Nuclear factor erythroid-2-related factor 2
HO-1: Heme oxygenase-1
MCAO: Middle cerebral artery occlusion
DMSO: Dimethyl sulfoxide
mNSS: Modified neurological severity score
TUNEL: Terminal deoxynucleotidyl transferase biotin-dUTP nick end labeling
TTC: 2,3,5-Tripenyltetrazolium chloride
SOD: Superoxide dismutase
MDA: Malondialdehyde
GSH: Glutathione
GFAP: Glial fibrillary acidic protein
Iba-1: Ionized calcium-binding adapter molecule
