# Supplementary material for: Dimethyl Fumarate and Monomethyl Fumarate Promote Post-Ischemic Recovery in Mice
Source: Transl Stroke Res. 2016 Sep 10;7(6):535–47. doi: 10.1007/s12975-016-0496-0 (PMC5065588; doi:10.1007/s12975-016-0496-0)
Supplement: Supplementary file 1 — (DOCX 123 kb) [file 12975_2016_496_MOESM1_ESM.docx]

**Supplemental Figure 1.**

**
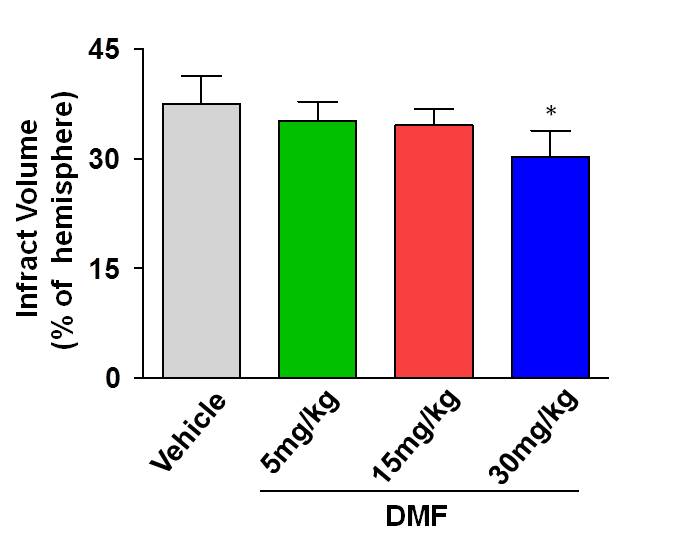
**

**Supplemental Figure 1. DMF protection against acute ischemic stroke is dose dependent.**

Quantitative analysis of infarct volume at 72h post MCAO ischemia-reperfusion injury.. Data are expressed as Mean ± SEM. *p<0.05 as compared to that in vehicle treated control group, n=8 in each group.

**Supplemental Figure 2.**


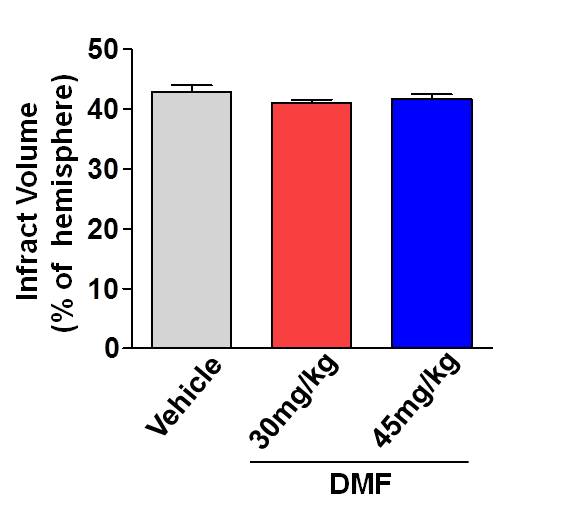


**Supplemental Figure 2. Cerebral infarct volume on day 1 post ischemia-reperfusion injury.**

At 24 hours following MCAO ischemia-reperfusion injury, the percentages of cerebral infarct remain unchanged with DMF 30 or 45 mg/kg body weight treatment (p=0.463). Data are expressed as Mean ± SEM, n=10 in each group.

**Supplemental Figure 3.**


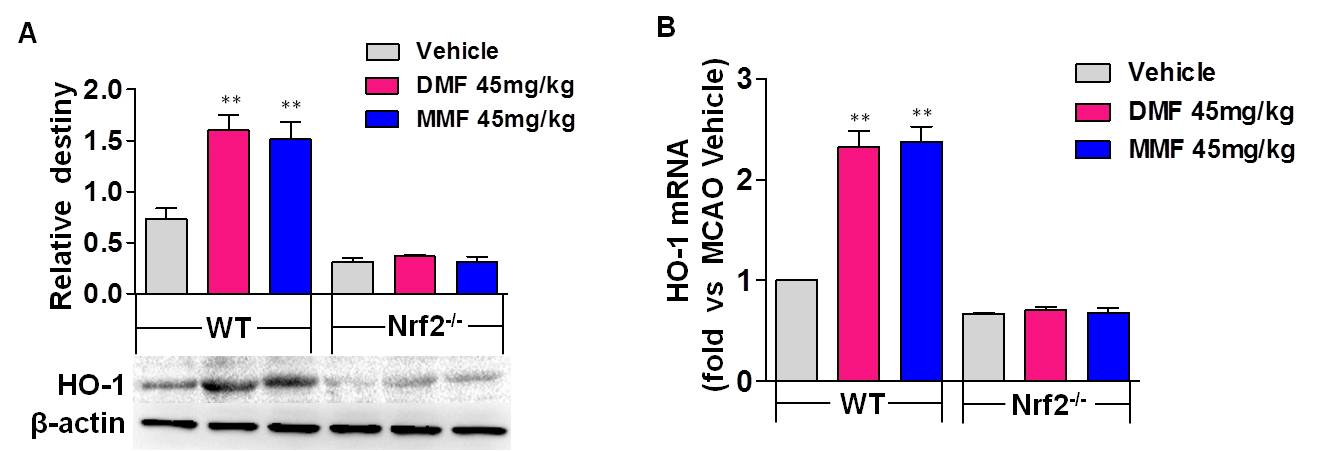


**Supplemental Figure 3. Nrf2 pathway is essential for DMF or MMF induced HO-1 expression.**

Levels of HO-1 protein (A) and mRNA (B) are increased in cerebral tissues from mice with MCAO ischemia-reperfusion injury upon DMF or MMF treatment. Absence of Nrf2 completely blocks the DMF or MMF induced HO-1 expression. Data are presented as Mean ± SEM, **p<0.01, as compared to vehicle treated wild type mice. The level of HO-1 protein was not different between DMF-treated Nrf2^-/-^ mice or MMF-treated Nrf2^-/-^ mice with vehicle treated Nrf2^-/-^ mice (p=0.226, p=0.969). There was no difference in the expression of mRNA between DMF-treated Nrf2^-/-^ mice or MMF-treated Nrf2^-/-^ mice with vehicle treated Nrf2^-/-^ mice (p=0.968, p=0.694), n=6 per group.
